# Mobilities of Older Chinese Rural-Urban Migrants: A Case Study in Beijing

**DOI:** 10.3390/ijerph16030488

**Published:** 2019-02-08

**Authors:** Yang Cheng, Mark Rosenberg, Rachel Winterton, Irene Blackberry, Siyao Gao

**Affiliations:** 1 Faculty of Geographical science, Beijing Normal University, Beijing 100875, China; ggaossiyyao@126.com; 2 Department of Geography and Planning, Queen’s University, Kinston, ON K7L 3N6, Canada; mark.rosenberg@queensu.ca; 3 John Richards Centre for Rural Ageing Research, La Trobe University, Melbourne, Victoria 3689, Australia; R.Winterton@latrobe.edu.au (R.W.); I.Blackberry@latrobe.edu.au (I.B.)

**Keywords:** rural-urban migration, older population, mobility, Beijing

## Abstract

Along with the rapid urbanization process in Beijing, China, the number of older rural-urban migrants is increasing. This study aims to understand how Chinese rural-urban migration in older age is influenced by, and impacts on the migrants’ mobilities. This study draws on a new conceptual framework of mobile vulnerability, influenced by physical, economic, institutional, social and cultural mobility, to understand older people’ experiences of migration from rural to urban areas. Forty-five structured in-depth interviews with older rural-urban migrants aged 55 and over were undertaken in four study sites in Beijing, using the constant comparative method. Results demonstrate that rural household registration (hukou) is an important factor that restricts rural older migrants’ institutional mobility. As older migrants’ physical mobility declines, their mobile vulnerability increases. Economic mobility is the key factor that influences their intention to stay in Beijing. Older migrants also described coping strategies to improve their socio-cultural mobility post-migration. These findings will inform service planning for older rural-urban migrants aimed at maintaining their health and wellbeing.

## 1. Introduction

Along with the rapid urbanization and increasing levels of population ageing in contemporary China, levels of older-age migration are increasing in Beijing, the capital city. This population ageing is exacerbated by a growing floating population in Beijing, which refers to internal movement of Chinese residents to places where they do not hold local household registration status (hukou) [1]. The hukou system was introduced in the 1950s as a method to regulate rural-urban migration, where moving between rural and urban localities required official permission. Each citizen was classified within an agricultural or non-agricultural hukou, with non-agricultural citizens receiving educational, health care, pension benefits not available to their agricultural, rural counterparts [2,3]. In 2000, the floating population was 2.56 million in Beijing, which included 63,700 older migrants. In 2010, the floating population increased to 7.04 million, among which the older migrants increased to 238,000 (3.39% of the total migrants and 9.7% of the total older population) [4,5]. The destinations of older migrants are consistent with the younger generation [6]. Nearly 90% of the total older migrants moved to the urban functional extension area and urban new developing area, which are newly developed areas in Beijing [7,8].

This older-age migration to urban areas can be somewhat attributed to the rapid socio-economic changes that occurred as a consequence of the Chinese economic reform at the end of the 1970s. During this period, many working-age Chinese relocated to megacities such as Beijing, either because they were educated and found better employment in megacities, or they migrated from rural areas to urban areas seeking employment opportunities. Consequently, parents of these working-age populations are also relocating in increasing numbers, in order to provide childcare, or access care and support as they age [6]. Existing studies have found that family factors play an important role in older people’s decision making around migration in China. When migration destinations, motivations and perceptions of Chinese rural and urban elderly are compared, family factors, such as caring for grandchildren, were the main migration motivation for the urban older adults, whereas searching for employment opportunities or receiving care and support from their children were the main factors for their rural counterparts [9,10,11,12,13]. Zhou [14] found that intention to migrate was generally low among Chinese older people, with availability of social networks, housing conditions and climate in the destinations having a significant impact on willingness to migrate. Consequently, this group have much synergy with the so-called zero generation or “transnational flying grannies” increasingly being reported in recent migration studies [15,16], where older populations migrate to host countries to provide childcare within transnational families. The vulnerability and invisibility of this ‘zero’ generation often results in a policy vacuum [17].

However, the vulnerabilities of older migrants increase with age and time, as their own health and care needs change through the life course, and their accessibility to health care in their new places of residence is rather scarce [17]. In relation to health and quality of life, older migrants are already a vulnerable group due to increased risk of poor physical and mental health, poor adaptation to changes in living environments, and (self-) exclusion from care and welfare services [18,19]. In China, rural-urban older migrants are even more vulnerable because of their relatively poor socio-economic status and accessibility to social welfare, low educational level and differences in lifestyle compared to their urban Chinese counterparts. Older people’s access to pensions and health care insurance are determined by where the person’s hukou is, and a lack of urban hukou (experienced by floating populations) restricts the ability of citizens to access health and welfare services in the place they live [2,3]. Since the 1950s, urban employees have enjoyed pension benefits, but rural residents and urban non-employed residents were excluded from such social welfare. In 2014, the Urban Residents Pension Plan and the New Rural Social Pension System were merged into a unified basic pension insurance plan for rural residents and non-employed urban residents. However, the benefit amount of this plan is very low, with a country average of 81 RMB (13 US dollars) per month, while the average household consumption per month in rural areas was 896 RMB in 2016 [20]. With regard to health care insurance, by the end of 2013, 95 percent of urban employed workers, non-employed residents and rural residents were covered by one of the medical insurance schemes, namely Urban Employee Basic Medical Insurance, Urban Resident Basic Medical Insurance and New Rural Cooperative Medical Insurance. However, even with these social welfare reforms, great disparities still exist between the urban and rural residents in various regions. Rural-urban older floating populations also face difficulties in accessing social welfare in the place where they were registered as they no longer live there. It is also more difficult to obtain reimbursement for hospitalization costs if they go to the hospitals outside of the regions where they are registered [21].

Literature on migration in both developed and developing countries mainly focuses on working age migrants [22,23,24,25,26]. The study of older migrants, especially rural to urban older migrants, however, is a relatively new field and remains marginal in migration studies, social gerontology, health and social geography. Researchers in English-speaking countries have undertaken studies on older-age migration since the 1960s. Existing studies focus on post-retirement moves, such as older migrants living in the northern US moving to the sun-belt areas [27]; older people migrating to live with adult children or relatives [28], and relocation to residential care facilities [29]. Many studies focus on the migration destinations, motivations, decisions and experiences of older people and the implications for ageing services and policy [30,31,32,33,34,35,36,37,38,39].

Consequently, mobility and vulnerability are two important concepts in the study of older migrants. Mobility is a key concept in migration research and transportation geography. It links the movements of a variety of things, including humans, ideas and objects, across different scales of moving in relation to forms of places, stopping, stillness and relative immobility [40]. One’s capacity to be mobile is dependent upon all sorts of social, political, cultural and economic contextual variables from “physical aptitude”, “aspirations to settle down”, other “existing technological transport and telecommunications systems and their accessibility” to “space-time constraints” such as the “location of the workplace” [41].

The critical turn underway in health and social geography emphasizes a shift from quantitative studies using statistics to qualitative studies focusing on personal, intimate and in-depth engagement with older people themselves [42]. Increasing attention has been dedicated to the social impact of amenity-led mobility in older age by looking at the degree of social relations in the local community and their social embeddedness [15,43,44]. However, from the Chinese perspective, the majority of existing research focuses on the migration of the labor force from inland China to the more developed east regions of China, by using statistical methods, national census data and social surveys to analyze migration patterns among older people [10,11,13,45]. Few studies have used qualitative methods for understanding older migrants’ mobility and migration decisions, and there has been no work exploring the intersection between older rural-urban migrants’ mobility and migration processes. It is important to understand mobility because the various forms of mobilities impact older rural-urban migrants’ willingness to migrate, experience of migration and their likelihood of staying in the new destination cities. Beijing, as one of the cities with the largest number of older rural-urban migrants in China, is used as a case study site within this research to explore how rural-urban migration among older Chinese is influenced by, and impacts on their mobilities. The purpose of this study is to provide a conceptual framework for rapidly developing countries, and demonstrate its value in exploring migration behaviours among older Chinese migrants. These results will shed light on behaviours relating to rural-urban migration among older people, which is important for future service planning to improve their health and well-being.

The following section reviews the literature related to mobility and introduces the conceptual framework of this study. The methods and results are discussed in the third and fourth sections, with conclusions and discussions presented in the last section of this paper.

## 2. Conceptual Framework

There are various forms of mobility, inclusive of but not limited to physical mobility, social mobility, and economic mobility. Mobility is not only the potential to be mobile but is also the ability to turn that potential into an actuality, which is influenced by physical, socio-cultural and economic factors [41]. People’s potential mobilities are constrained by the particular space-time prism they belong to, which is defined by their class, identity, income or other characteristics. Mobilities are also socially differentiated, and are linked to a wider context of established societal norms, codes of conduct, belief systems and ideologies [46]. Insufficient mobility may threaten social inclusion because of reduced accessibility to opportunities, services and social networks [47]. People may have little choice because of the sorts of mobile societies they must move within. Researchers have also sought to understand the relationship between mobility and privilege, politics and exclusion [48]. Some studies have focused on the inequalities and exclusion of mobility practices and spaces among marginalized groups to understand the diverse experiences particular social groups may have of mobility [49,50]. These studies show that mobilities have a significant impact on people’s decision to migrate, and behaviors associated with migration, as well as their ability to adapt.

In terms of vulnerability, Bustamante et al. [51] identifies “mobile vulnerability” as a social condition where the human rights of migrants are violated. Multiple vulnerabilities associated with mobility were identified among migrants, which were associated with language and cultural barriers, ethnicity, race or low socio-economic status.

**Figure 1:**
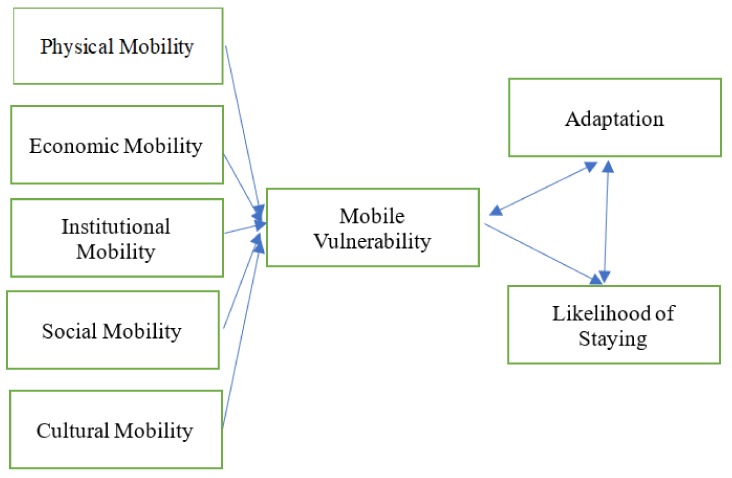
The conceptual framework of mobile vulnerability.

Based on the review of the literature, this study will investigate the physical, economic, institutional and socio-cultural aspects of mobility based on the forms of mobility and its consequences. We are proposing this conceptual framework as a way to understand the vulnerabilities that older rural-urban migrants face, and the role of agency in developing coping strategies that help them adapt to the new living environment (Figure 1). Physical mobility refers to the physical capability of an individual to migrate and adapt to the new living environment. Economic mobility refers to the financial affordability of migration and cost of living in the migrant’s destination. Institutional mobility refers to mobility that is affected and restricted by policies and regulations. For example, limited social welfare benefits for older rural-urban migrants at the destinations restricts their mobility. Social mobility refers to mobility affected by one’s social connection and embeddedness within the area of origin and destination, while cultural mobility refers to one’s mobility affected by cultural norms and habits within society. The mobile vulnerabilities that older migrants experience, and their adaptive capacity, influence their likelihood of staying in their new destinations. The frameworks developed in developed countries, however, do not take into account the kind of issues that exist in rapidly developing countries such as China. Chinese older people face various challenges associated with rural-urban migration, such as access to social welfare benefits, the household registration system, psychological adaption and the availability of care resources [9]. In this study, we will conduct empirical research to test the conceptual framework proposed in Figure 1.

## 3. Data and Methods

**Figure 2:**
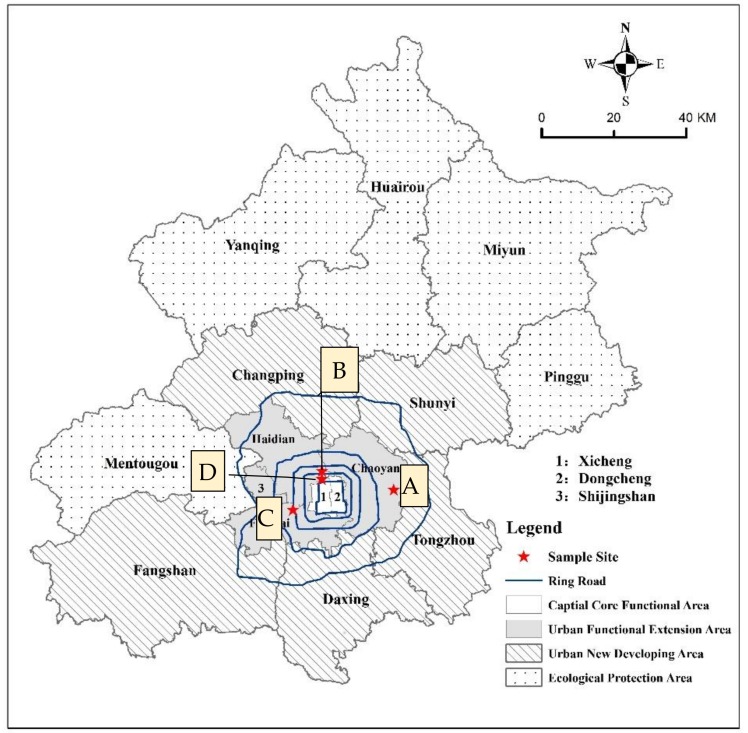
The location of the case study sites.

To answer the research questions, 45 structured in-depth interviews were conducted with older people aged 60 and over in four sites in Beijing, including two residential quarters, the campus of Beijing Normal University and a park (Figure 2). All the four sites are located in the urban function extension area of Beijing where most of the older migrants are located. More detailed information on the four sites are introduced below. The demographic characteristics of the participants are listed in Table 1. Participants were aged between 55 and 86, and had been living in Beijing for two months to 30 years.

Beichenfudi community (A) is located in Chaoyang District. Established in 2011, it was a social housing project with a relatively low price for rent and sale. The total number of households in this community is 3891, with 15,000 permanent residents. The population aged 60 and over is 1950, which accounts for 13 percent of the total residents. 

Beitucheng Park (B) is a public park built on the historical site of the old city wall. It provides open space for recreation, ecological protection and emergency shelter. There is fitness equipment available in the park. It is easily accessible by public transportation and attracts many older people who live close to the park to exercise and carry out various social activities, such as chorus, dancing and Tai Ji in the park.

Chang’anxincheng community (C) is located in Fengtai District. It was one of the first social housing projects built in Beijing in 2002. The total number of permanent households is 4700 with 12000 permanent residents. The population aged 60 and over is 2100, which accounts for 17.5 percent of the total residents.

The campus of Beijing Normal University (D) is located in Haidian District. The residents include the families of university faculty, staff members, and retirees. Many social activities including singing group, calligraphy and handcrafting are also organized. Some contract workers who are responsible for cleaning and gardening on campus are rural- urban migrants, including some older migrant workers.

Convenience sampling was conducted in the open space of the four study sites, which are popular gathering places for Chinese older adults. Six research assistants went to the four study sites to interview participants during the daytime in April 2017. Interviewers approached potential participants within these public areas to ascertain if they met the age criteria (55 and above), had migrated from rural China to urban Beijing, and still held rural household registration. If they met these criteria, they were provided with information about the study and invited to take part in an interview. Interviews covered demographic information (age, marital status, income, occupation before migration, level of education, length of residence in Beijing, living arrangements), reasons for migration, impact of migration on health and quality of life, level of adaptation and adaptation strategies, patterns of health care utilization and plans to stay in Beijing. Ethical approval was received from the La Trobe University human ethics committee (HEC17/005).

Interviews were audio-recorded with participant permission and transcribed into Mandarin and English. The analysis of the data is based on the constant comparative method [52,53,54]. Transcripts were open-coded and the physical, economic, institutional, social and cultural aspects of mobility were summarized based on the open-codes. Following the interview questions and the themes, how older people adapt to the new environment and their likelihood of staying in the destination were also analysed. Transcripts were coded deductively by the first author and two research assistants in relation to the interview questions. This coding was then ratified by other co-authors, and discussed in relation to key themes.

## 4. Results

The majority of participants are married, and have lived in Beijing for more than one year. Some had lived in Beijing for an extensive period (over ten years), which suggests that while they had migrated, they were to some extent ageing in place. The majority also had less than nine years formal education, and 29 out of the 45 participants receive less than 1890 RMB per month, which is the minimal wage in Beijing in 2016. Two thirds of the participants were farmers before they migrated to Beijing and one third were employed before their migration. For the younger participants, caring for grandchildren was the major reason for migration to Beijing, while some migrated to Beijing for economic reasons as they are still capable of making money by doing physical work. In contrast, the major reason for the oldest-old to move was to live closer to their family members for elderly care and health care.

There was consensus among the five authors that mobile vulnerability was a prominent theme. The vulnerability experienced by rural-urban migrants related to various forms of mobility, including physical, economic, institutional and socio-cultural components of mobility. Their coping strategies to adopt the new living environment and their willingness to stay in Beijing were also discussed.

### 4.1. Physical Mobility

From the individual perspective, the physical mobility of older migrants changes through the life course. The change of mobility influences the reasons for migration. The older migrants move due to economic driving forces or to provide childcare for their grandchildren when they are physically capable. Their purpose for migration changes to seeking care support from their adult children when their physical mobility declines. Some older migrants reported that their willingness to stay in Beijing depended on their health status and mobility. The two quotes below reflect the various motivations for migration related to physical mobility.

*My son is still young, not married yet. I am working here. My son needs a large sum of money. Young men need a lot of money to get married in rural areas. (B9, 65 years old, living in Beijing for less than a month).*

*I am 87 this year. I came here for [better] health care. I could die in half an hour when the [heart] disease occurs. Everything is expensive here, but I have no choice. (B6, 87 years old, living in Beijing for one year).*

It is well recognized that access to transportation improves people’s physical mobility. The increasing use of private cars and high-speed trains made it much more convenient for the older migrants to travel between their hometown and Beijing. This increase in physical mobility somehow increased their willingness to migrate. However, participants also mentioned that this improved access to public transportation was contingent on their adult children to offering rides or financial support for travel expenses, and this vulnerability relating to physical mobility and financial affordability limited their ability to travel.

*I was from Yanqing [one of the rural districts of Beijing]. I have two daughters. One lives in Yanqing and the other lives in the city. I live with their families in turns. Both of them have cars. Every time I move, they drive me to the other daughter’s home…If I don’t have the two daughters, I would die already. (C8, 80 years old, living in Beijing for 20 year).*

Physical mobility is also linked to the mobility older migrants experience within their living neighborhood. Rural-urban older migrants are more vulnerable compared to their local counterparts due to their unfamiliarity to the new living environment, inadequacy of spare time, loneliness, and language barriers, which limit their physical mobility. Even though some participants mentioned the free bus they can use in Beijing, few of them actually used the public transportation alone due to limited spare time or lack of familiarity with their living environment.

*I was quite upset during the first two years I was here. I felt I was a stranger in a strange place. I did not know the people here and I was not well educated. I did not dare to go anywhere…People come here [park] to dance or do some exercises. I don’t have time. I just come out for a walk. Then I get to go back home to cook, care for the kid, and clean the apartment. (A2, 62 years old, living in Beijing for six years).*

### 4.2. Economic and Institutional Mobility

Post-migration, participants demonstrated a strong sense of vulnerability related to their economic status. In some cases, older migrants intended to temporarily migrate to Beijing and planned to move back to their hometowns because of their vulnerable economic and institutional mobility. Many of them migrate to provide care support for their grandchildren. Low income, small living space, high living expenses and health care costs limited their mobility, which resulted in high financial dependency on their adult children’s family. When the grandchildren grow up, they would be forced to move back to their hometown due to these reasons.

*I don’t want to come here. I don’t want to stay here for one [more] day, (laughing…). This is not my home, and I don’t have money. I have to ask for money from my daughter. My daughter has limited income. It is not like I can buy what I want, right? If it is my own money, I can buy what I want to buy, and I can do what I want to do…. But I understand, I come here to look after my grandchild. (A2, 62 years old, living in Beijing for six years).*

*Ah, nowadays, the kids need to be picked up and sent to school even when they go to primary school. I cannot think anymore, and I don’t want to think more about it. Sometimes, it makes me upset to think about these things. When the kids grow up, I am old, and I cannot stay here anymore, then I have to go home. I cannot do anything when I go home and I will be upset again. But to stay here, it won’t work. My grandson needs space. Right? It is not to say my daughter doesn’t show her filial respect to me. They are not able to do that, how can they show their respect? They work hard and are tired. They only get this small space, right? (A2, 62 years old, living in Beijing for six years).*

The social welfare that rural-urban migrants can access ties to their hukou instead of their living place, which affects their institutional mobility. Rural older adults are less likely to have access to state supported social security benefits in the rural system and more likely to rely on support from their adult children. Consequently, many participants reported that they intended to live in Beijing on a temporary basis to provide childcare while they were physically capable, but would eventually like to move back to their hometown because they did not have the hukou to access social welfare benefits in Beijing. This vulnerability in economic and institutional mobility experienced by the rural older migrants is obvious, and influenced their sense of belonging in Beijing and willingness to stay.

*Yes, I come here just for providing support to them. You don’t have money, and you don’t own housing. There is no living space for you. When the kid grows up, he needs space. [It’s] no better than going back home, to live in my own small house (A2, 62 years old, living in Beijing for six years). *

*Here I don’t receive any health care benefits. In my hometown, I can get 50% reimbursement for medical cost. Now we have the rural cooperation medical plan in rural areas. My husband was retired from the company so he can receive 90 percent reimbursement for his medical cost. (A3, 63 years old, living in Beijing for four years).*

The Beijing municipal government, however, is making efforts to gradually improve social welfare benefits for the older migrants. For example, older migrants who aged 65 and over and have lived in Beijing for more than six months can apply for an elderly bus card, which provides them with free access to the bus system in Beijing.

*The bus is free for the older people by using the elderly card. It is convenient. The older people can apply for such card if they stay long enough. I applied for the elderly card here, and I have been here for nine months. It is long enough. We applied for it in the community by providing my daughter’s ID card and certificate for residence… It is convenient for the older people to live here once they have the medical card and bus card. (C1, 67 years old, living in Beijing for one year).*

### 4.3. Socio-Cultural Mobility

Along with the physical, economic and institutional vulnerabilities that older migrants experience related to mobility, socio-cultural norms also have impacts on older migrants’ mobility and vulnerability. For rural-urban older migrants, a strong sense of filial piety and family ties associated with traditional Chinese culture still plays an important role in decision making relating to elderly care. As older migrants’ physical mobility decreases with age, they also face challenges associated with financial security and health status. They are more likely to seek family support by moving to Beijing to live with their adult children, due to the inadequacy of care support in their rural hometown.

*…I moved to live with my son. I stay where my son lives… (A4, 69 years old, living in Beijing for seven years).*

*When I get old and I don’t have self-care ability in the future, who will care for me? It is impossible for my children coming back to care for me if I move back to my hometown…It is all right, my son and daughter are living here. I wouldn’t move here if they don’t live here. (A10, 73 years old, living in Beijing for five years).*

However, the experiences of co-residency with their children’s family were not always as enjoyable as they expected. Some older migrants talked about conflicts in the family when they lived with their children, which influenced their perceived experience of migration.

*…it is not I am willing or unwilling to migrate. I have to come even if I am unwilling to do so. There is no choice. Of course, I was not used to live here when I first moved here. I am not used to live with my son and daughter in law. The living space is small, and the living habit is different. It is not convenient. The housing is different from the rural area. I am a total stranger and unfamiliar with the place and people. There is no choice and I am not willing to come. It is not an obligation, but I also have the responsibility to support them (my children). (A8, 66 years old, living in Beijing for six years).*

Older people’s authority within one’s household in the traditional Chinese culture is challenged in modern society, especially for the older migrants. The implementation of the one-child policy (a population planning policy introduced in China in the late 1970s and formally phrased out in 2015, to limit the great majority of family units to have only one child each) enhances the worries of the older migrants about the care burden on their child. Quite a few participants mentioned that their willingness to stay in Beijing was dependent upon their children’s decision.

*My son wants me to stay here. I have only one son, and no more child. I am the first cohort who has the one-child, post 80s, right? I am not thinking about going back home. It is so lonely to go back, and my son is going to be worried about me, right? His father has passed away, and I stayed in the hometown by myself, for ten years! (D3, 62 years old, living in Beijing for ten months).*

*The housing in my hometown is not sold yet. If it is too much burden for my daughter in the future, we will go back home to move into residential care facilities. If we move to the residential care facility in Beijing with our little pension, we don’t think we can go to a good one. We will take my daughter’s advice. (D6, 64 years old, living in Beijing for thirteen years).*

Cultural factors also impacted their willingness to remain in Beijing. For example, one older migrant reported that his willingness to stay in Beijing was impacted by the different cultural norms associated with funerals, and he wished to move back to his hometown to follow his family’s burial traditions.

*I don’t want to be cremated here. I want to rest in a coffin and be buried with the ancestors together. (B4, 70 years old, living in Beijing for twenty years).*

Social exclusion and loneliness of older migrants is shown to vary greatly, depending not only on individual characteristics (e.g., age, health, educational level and length of stay in the destination), but also on environmental circumstances (e.g., characteristics of the residents in the neighborhood and availability of social meeting places). Some participants mentioned that they were busy with childcare and household chores, which left them little time to build a social network or attend in any social activities in the new living environment. Although older migrants are vulnerable due to their relatively low socio-economic status in Beijing and high dependency on their children’s family, some migrants manifest agency and develop strategies to cope with the real and potential vulnerabilities. They put efforts in building a new social network through caring for the grandchildren.

*There are many older people coming here to care for the grandchildren. We get in touch with others. Sometimes, we all come out with the kids, and we get together to chat a little bit. (C1, 67 years old, living in Beijing for one years).*

Personal attitudes towards population ageing also impacted on decisions relating to migration. Active ageing is beneficial for keeping one’s mobility both physically and socio-culturally and it was mentioned by some older migrants when explaining their reasons for migration.

*My children all moved to cities. I don’t want to stay at home and I am tired of doing the farm work. I moved to Beijing for work. I am a gardener now. I have less workload and make more money. As a farmer, doing physical work is a way to keep fit. The other good thing is that I got to live in the capital [city] and visit many places of interest and parks. (D10, 61 years old, living in Beijing for three years).*

The new social connection with the local residents or other migrants helped the older migrants feel more sense of belonging within their communities. Older migrants also received support from their families and stayed connected with the home environment through the use of communication technology, such as cell phones and WeChat (a Chinese app that can make video calls, Tencent Co., Shenzhen, China), which decreased the psychological distance between Beijing and their hometowns. Some had adapted to their new life post-migration, after a longer period of residence in Beijing.

*My health [status] has improved after the migration. I have set up my routine here, which I did not have when I was in my hometown. Just doing my farm work, and having meals after finishing the work. Here is different. I eat and come out for a walk on a fit schedule. When I first came here, my health [status] was not so good as before. I was not used to moving here. My health status was even worse than I was at home. Gradually, I figured out that adapting to the new life is the way I must go. (C5, 66 years old, living in Beijing for seven years).*

The willingness to stay ties to the physical, economic, institutional and socio-cultural aspects of mobile vulnerability that older migrants experience. The adaptation process reflects the role of agency in developing coping strategies for meeting the challenges related to migration, which also affects older migrants’ mobile vulnerability.

## 5. Discussion

This case study on mobility among rural to urban older migrants demonstrates how the proposed conceptual framework is valuable for future study. Certain elements of our findings confirm previous results of other studies. Studies conducted in Guangzhou and Dalian showed that many older migrants moved to cities to provide care for the grandchildren and help with the housework for the adult children [55]. They remain highly dependent on their adult children in the new environment in terms of finances, housing, social life and prospects to return. The restricted social network within the families generates feelings of loneliness and social exclusion in the new living environment and increases their social vulnerability [56]. These findings were also reported by studies on the “zero generation” in the transnational migration among international migration in the older age [15,57]. However, in the Chinese context, many older migrants mentioned that they can manage to live with their adult children if their children’s family needs their support, even though they have faced difficulties in adapting to the new living environment. This is the representation of responsibilities of older parents in the household according to the traditional Chinese culture even though they face various forms of mobile vulnerability post- migration. The older parents offer help for childcare and housework when they are the young-old and the adult children provide care support for them in compensation as their parents get old and need family care [58].

Some of our findings, however, show differences compared to other studies. For example, Meng et al. found that most of the older migrants were satisfied with the physical and socio-cultural environment, the neighborhoods in which they live and health care services in Beijing [11]. More than 70 percent of the older migrants were willing to stay in Beijing. The findings from Meng et al. differ from the results of this study, where most of the participants discussed their dissatisfaction with the high cost of health care services in Beijing. Many of them reported they would plan to move back to their hometowns as they grow older [11]. One potential explanation of this difference is that the participants in the study conducted by Meng et al. were relatively highly educated, had relocated as a couple and had urban household registration, which is different from the participants as rural-urban older migrants in this study.

Many rural-urban older migrants are a vulnerable group due to their invisibility, especially for those who are unregistered. The increase in older migrants in Beijing brings many challenges for the megacities to provide care support for the older migrants. Although governments are making an effort to introduce social welfare reform to provide a safety net for older people with rural household registration, health and social benefits are currently tied to the household registration [59]. The older migrants’ relocation to urban areas does not automatically entitle them to old-age pension benefits, and care policies and formal arrangements do not address the particular needs of this population [60,61]. A study by Liu and Feng (2015) suggests that within megacities such as Beijing and Shanghai, there are more stringent standards for granting hukou due to a desire to attract wealthier, more educated migrants. Consequently, older migrants are often concentrated on the peripheries of cities, with limited ability to access social benefits, services and welfare. During their reconstruction of identity in the new urban environment, the materials and power relations structured in the urban environment make the older migrants marginalized in communicating with the local people and socio-culturally embedded in their destinations. Improving the community services in the megacities will help the older migrants reconstruct their social support network and promote their communication with the local people [3].

While the length of time that some of the participants had been resident in Beijing suggests that to some extent they are ageing in place, our findings indicate that Beijing was not the place where they were likely to age with a high quality of life. Our study demonstrated that many older migrants plan to move back to their hometown, whether they are willing to do so or they are forced to go back due to the mobile vulnerability they experience. Consequently, care for older people in rural China will face many challenges, as a result of the proportionally larger number of elderly residents and the lack of options for care [61]. To address the increasing future challenges and improve older migrants’ vulnerabilities, actions should be taken at the macro, meso and micro levels. At the macro level, national policies on social welfare should continue to be improved to mitigating older migrants’ vulnerabilities. Policies on the provision of social welfare benefits should take into account the needs of older migrants and be universal to cover the older population based on their age instead of their hukou. The meso level captures community and family networks and resources to support care and other needs in older age. For example, at the community level, Beijing municipal government requires the migrants to register at the street office for temporary residence permits. In the future, older migrants’ need can be evaluated at the community level after their registration. The provision of community services can also be tailored based on the individual needs of older migrants. The micro level refers more specifically to individual factors, such as improvement and adaptation of the migration experience, socio-economic level, health condition, and active attitude towards ageing of older migrants. These findings suggest that social policies should address older migrants’ needs in a differentiated manner and target the most vulnerable groups in specific ways [15].

The small sample size of this study is a limitation. Additionally, the recruitment strategy used, where participants were recruited in public space, suggests that the participants interviewed may be more physically and socially mobile than other rural-urban migrants. As a result, this study cannot be considered representative of rural-urban older migrants. However, the findings of this study help understanding the mobility and vulnerability of this distinct and increasing population group. 

## 6. Conclusions 

This study draws on a new conceptual framework of mobile vulnerability, influenced by physical, economic, institutional, social and cultural mobility, to understand older people’ experiences of migration from rural to urban areas. The rapid socio-economic growth after the economic reform in China in the late 1970s has affected people’s mobility in various ways. Physical mobility improves with better access to public transportation system. The change from the centrally planned economy following the Soviet Union model to the market oriented economy for improving the productive efficiency since the late 1970s has created many job opportunities in urban China, which improves economic mobility for the migrants. 

However, the implementation of the one-child policy for family control in the same period as the economic reform has decreased both household size and family care resources. These outcomes affect people’s mobility in institutional ways, together with the different rural and urban social welfare benefits tied to the household registration. A large number of labor workforce migrate from rural China to urban China either for jobs or for education and stay in urban China after receiving education. The increasing migration of working-age adults to urban regions has dramatically altered the traditional patterns of co-resident living arrangements and intergenerational support for rural older people. As a result, the weakening of traditional patterns increases the numbers of older adults living alone, and decreases quality of life and support for older parents living in rural areas [62].

Consequently, the older parents of the young migrants also move to urban areas. The majority of them migrate for care resources, either providing care for their grandchildren or receiving care from their adult children. A small proportion of the older migrants move for economic factors. The traditional cultural norms are gradually changing to adapt to these rapid socio-economic changes in China, such as a lessened expectation on instrumental care support from adult children due to increased distance or busy schedules, and the change relating to older people’s level of authority in the household, due to the vulnerable socio-economic status of the older migrants who are co-residing with their adult children. These socio-cultural changes enhance the mobile vulnerabilities that the older migrants experience. Meanwhile, older migrants have limited access to the pension and health care insurance and enjoy less welfare benefits in their urban destination due to the lack of urban hukou. The vulnerability in institutional mobility created by the hukou system also affects their economic mobility.

While this study focuses on China, many aspects of the research, such as the implication of promoting active ageing at the individual level, developing community services at the local level, and providing a safety net at the national level to improve quality of life for the older migrants, may also be relevant, in the context of mobility, to other countries. The results help provide evidence for future planning and policy making. In the future, more research on the structural improvements and adaptation for older migrants living in megacities in China is required.

## Figures and Tables

**Table 1:** The information on the participants.

Characteristics	Number of Participants	Characteristics	Number of Participants
Age		Length of Stay	
55–59	6	Less than 1 year	6
60–69	25	1 to 5 years	21
70–79	9	Over 5 years	17
80+	5	N/A	1
Gender		Residential community	
Male	25	Beichenfudi (A)	10
Female	20	Mudanyuanxili (B)	12
Marital Status		Chang’anxingcheng (C)	9
Married	34	Beijing Normal University (D)	14
Widowed	9		
Single	1		
N/A	1		
